# Anthropometric differences between twins at birth and their association with later cognitive performance

**DOI:** 10.1111/jcpp.70170

**Published:** 2026-05-23

**Authors:** Robert Eves, Marco Deppe, Christian Kandler, Bastian Mönkediek, Sakari Lemola

**Affiliations:** ^1^ Department of Psychology and Sports Science Bielefeld University Bielefeld Germany; ^2^ Department of Psychology, Lifespan Health and Wellbeing Group University of Warwick Coventry UK; ^3^ Department of Psychology, Institute for Psychology University of Bremen Bremen Germany

**Keywords:** Birthweight, head circumference, twin, longitudinal, cognition

## Abstract

**Background:**

Past studies indicate significant associations between birth anthropometrics, such as relative birthweight or head circumference, and later cognitive performance. Using twin data, we investigated whether these associations are confounded by genetic and environmental factors. Additionally, we determined whether the association of within twin differences in birth anthropometrics and cognitive performance is nonlinear or moderated by factors such as zygosity or age at assessment.

**Methods:**

Longitudinal data from over 2000 twin pairs from the German TwinLife study were analysed using the co‐twin control method. Cognitive performance was assessed at two waves, with median ages of 12 and 18 years, with *z*‐standardised scores based on wave and age. Differences between twins in birthweight and head circumference *Z* scores were calculated using Fenton's size at birth chart, based on health records at birth.

**Results:**

Within twin differences in relative birthweight were associated with differences in cognitive performance at Wave 1 (*β* = .08, *p* = .001) and did not differ by zygosity (*β* = .03, *p* = .560). However, this association was not significant when reducing the sample to twins with valid data at both waves (*β* = .05, *p* = .187). Testing for nonlinearity or for moderation by age at assessment did not improve model fit. Within twin differences in head circumference were not significantly associated with differences in cognitive performance in any analyses (smallest *p* = 0.365).

**Conclusions:**

After controlling for confounding due to genetics or the shared environment, conditions in the womb, as indexed by relative birthweight, is associated with later cognition. This association is not stronger in younger individuals.

## Introduction

There are numerous studies investigating the long‐term consequences of infants being born at neonatal risk (Eves, Mendonça, Baumann, et al., 2021; Eves, Wolke, Spiegler, & Lemola, 2023; Johnson & Wolke, 2017; Twilhaar et al., 2018). In particular, there is strong evidence that infants born with a lower gestational age (preterm birth, born <37 weeks gestation), a lower birthweight (LBW, birthweight <2,500 g), a lower relative birthweight (i.e. small for gestational age, SGA, birthweight percentile <10%), or a smaller head circumference, have lower cognitive performance in childhood and adulthood in comparison to their term born, normal birthweight (NBW), appropriate for gestational age (AGA), or larger head circumference peers (Eves, Mendonça, Bartmann, & Wolke, 2020; Freire et al., 2023).

However, the majority of studies in this field compared infants of differing neonatal risk who are also from different families, raised in vastly different environmental settings. Environmental factors that may influence children, such as poverty, may not only relate to differences in birth outcomes, but also differences in cognitive outcomes of children (Cubbin, Kim, Vohra‐Gupta, & Margerison, 2020), leading to a spurious association. In addition, given differing genetic susceptibility to cognitive performance and a genetic risk component to being born preterm or at LBW, it is possible, or even probable, that the association between neonatal risk and cognitive performance is partly confounded by genetic effects (Husby, Wohlfahrt, & Melbye, 2023).

Considering possible genetic and environmental confounding is important when one is interested in the accurate and early identification of infants at risk for lifelong issues. One way of addressing this concern is to consider differences between twins, and in particular among monozygotic twins (MZ). MZ twins reared together are genetically identical and are also matched on gestational age. Additionally, their environments are typically far more similar than two randomly matched children in the population (Edmonds et al., 2010). Utilising data on MZ twins, numerous environmental factors that act to make them more similar are naturally controlled for and thus superior to statistically controlling for numerous covariates as is traditionally done when using population samples (McAdams, Rijsdijk, Zavos, & Pingault, 2021). Crucially, MZ twins can differ in anthropometrics such as their birthweight and their head circumference (Raznahan, Greenstein, Lee, Clasen, & Giedd, 2012). Thus, twin studies, and MZ twins in particular, offer the unique opportunity to investigate the association between anthropometrics at birth and later cognitive performance while controlling for genetic effects and providing a stricter control for numerous potentially confounding environmental factors, such as familial socioeconomic status or maternal education shared by twins reared together.

As MZ twins have the same genetic growth potential, differences in anthropometrics at birth can be thought to be attributed to differences in the nutrition and oxygen provided by the placenta(s) to each twin and thus measuring the deviation from growth potential (Groene et al., 2022). Therefore, if differences in birthweight between twins is predictive of later differences in cognitive performance, it provides stronger evidence for causality regarding conditions in the womb and cognitive performance relative to studies using general population samples. Empirical evidence from one twin study found a 500 g increase resulting in a 2 IQ point gain (Raznahan et al., 2012). Arguably, using an anthropometric measure which is adjusted for gestation is superior to using raw values of birthweight measured in grams. For example, a 100 g difference may be relatively insignificant for term born twins but may be a substantial difference between very or extremely preterm twins. When instead using relative measures, a 1 SD increase in birthweight z‐standardised scores was associated with a 15% increase in math performance (Torche & Echevarría, 2011) while having a birthweight 20% higher was associated with 0.10 to 0.24 SD reduction in attention problems (Groen‐Blokhuis, Middeldorp, van Beijsterveldt, & Boomsma, 2011). Overall, results currently indicate that being the larger twin at birth is advantageous for later developmental outcomes.

While past studies indicate that relative birthweight remains significantly associated with later development after controlling for genetic factors, further questions still remain. First, instead of using weight, head circumference at birth may offer superior prediction of later cognitive outcomes as a proxy indicator of brain growth (Flensborg‐Madsen, Eriksen, & Mortensen, 2020; Jaekel, Sorg, Baeuml, Bartmann, & Wolke, 2019). Additionally, the association of anthropometrics at birth with later cognitive performance may demonstrate a nonlinear association. For example, in nationally representative samples, increasing relative birthweight was only associated with increasing IQ performance up to the 69th percentile of birthweight, with a plateauing effect afterwards (Eves et al., 2023). Thus, one may not expect cognitive differences between twins, even when they have differing birthweights, if both have a birthweight percentile above a relatively high threshold.

Furthermore, relative birthweight's association with cognitive performance may be moderated by additional factors. For example, in general population samples, it has been suggested that being born SGA may be more of a risk factor to cognitive performance when one is also born more prematurely, as it is then thought to be more indicative of foetal growth restriction instead of being constitutionally small (Shah & Kingdom, 2011). However, empirical evidence from population and cohort studies has yet to support this hypothesis (Charkaluk et al., 2012; Eves et al., 2020, 2023). Moreover, it is thought that foetal growth restriction earlier in the pregnancy is more strongly associated with lower cognition relative to growth restriction within the last trimester (Chen, Liu, Zhou, et al., 2024). Thus, when a twin is born with a lower relative birthweight at an earlier week of gestation, they are more likely to have been exposed to the potentially more pernicious effect of early growth restriction. This would result in the association between relative birthweight and later cognition to be moderated by week of gestation, with the association being stronger in twins born at an earlier week of gestation.

Finally, based on cross‐sectional evidence, the association between anthropometrics at birth and cognitive performance was thought to diminish into adulthood (Eryigit Madzwamuse, Baumann, Jaekel, Bartmann, & Wolke, 2015). On the other hand, in one relatively small longitudinal analysis, SGA's association with cognitive performance throughout development appeared to show little to no diminishing association across the first 26 years of life (Eves et al., 2020). Whether this longitudinal effect will be replicated in larger samples using data from twins is currently unknown. In sum, it is currently not well understood whether the association of anthropometrics at birth with cognitive performance is consistent across different anthropometric measures (i.e. birthweight and head circumference), nonlinear, moderated by either gestational age, or moderated by age, especially after stricter controlling for genetic and environmental confounding. To address this, we use data from the TwinLife study, a German twin family panel study, to investigate three main research questions:

1. Within twin pairs, do differences in birth anthropometrics, such as weight and head circumference, linearly or nonlinearly predict later differences in cognitive performance?
2. Does the association of within twin pair differences in birth anthropometrics and cognitive performance diminish when both twins are born with relatively high birth anthropometric values?
3. Does zygosity, gestational age, or age at assessment moderate the association of within twin pair differences in birth anthropometrics differences and later cognitive performance?

## Data and methods

### Sample and procedure

TwinLife is a German, longitudinal study comprising same‐sex twins (Diewald, Riemann, Spinath, et al., 2016; Lang & Kottwitz, 2020). In the first wave in 2014/15, 4 cohorts of approximately 1000 pairs of MZ and dizygotic (DZ) twins were recruited. They were on average 5, 11, 17, and 23 years of age at this first measurement wave, with the resulting sample thought to be nationally representative (Lang & Kottwitz, 2020). Ethical approval was provided by the ethic committee of the German Psychological Association (DGPS, protocol numbers: RR 11.2009 and RR 09.2013) (Lang et al., 2020) while funding for the study was provided German Research Foundation (DFG, Grant Number 220286500). The preregistration for this specific analysis can be found on the OSF. The only significant deviation from the preregistration is that we have limited the current analysis to only cognitive performance and will test for mental health differences in a future analysis/publication – https://osf.io/j43k8/?view_only=b0b873688327434ea8dc7febd321486a.

### Birth anthropometric data and Zygosity

Gestational age (in weeks), birthweight (to the nearest 5 g), and head circumference (to the nearest 0.5 cm) data were taken from the ‘U‐Heft’ where doctors report the health of the infant immediately after birth. Additionally considering the participants' sex, the Fenton reference was used to determine birthweight z‐standardised scores (BWZ) and head circumference *z*‐standardised scores (HCZ) (Fenton & Kim, 2013). The Fenton reference uses data from multiple countries to develop an international chart that identifies the average birthweight (or head circumference) at each gestational week. Participants with implausible BWZ or HCZ values lower than 0.003% (*z* score < −4) or >0.998% (*z* score >4) were not included in analyses, as these likely reflect reporting errors (Chou, Roumiantsev, & Singh, 2020). Twin pairs with differences in their reported gestational age were also removed, as this likely reflects a reporting error. As is standard in twin analyses, we then calculated each twin pair's mean BWZ and HCZ (x¯‐BWZ_Twin_ and x¯‐HCZ_Twin_) as well as the within‐pair difference (Δ‐BWZ_Twin_ and Δ‐HCZ_Twin_), which are then subsequently used as the main independent variables in all analyses. Zygosity was determined using physical similarity questionnaires, administered in the first wave, which has been subsequently verified by genotyping a subsample of the twins (Mönkediek et al., 2019).

### Cognitive performance

For the present analysis, we used cognitive performance data from two assessments in Wave 1 (2014–16) and Wave 4 (2020–22), 6 years apart. In Wave 1, if participants were aged under 10 years old, cognitive performance was measured using the revised Culture Fair Test (CFT 1‐R) (Weiss & Osterland, 2012). The test comprises of three subtests: figural reasoning, figural classification, and matrices.

For participants aged over 10 years old, at both Waves 1 and 4, the same cognitive tasks were administered. These were figural reasoning, figural classification, matrices, and reasoning from the CFT 20‐R (Weiß, 2018). Further information regarding the TwinLife cognitive testing procedure has been previously published (Gottschling, 2017).

Initial analyses only used cognitive data from Wave 1, adjusting for the varying age at assessment within each subtest so that there was no main effect of age on cognitive performance. For the under 10 and over 10 cognitive tests, we *z*‐standardised and then combined the subtests so that the overall mean was 0 with a standard deviation of 1 for both tests (Table 1). For longitudinal analyses, we analysed twin pairs with full cognitive data at both waves, again adjusting each subtest for age at assessment and then standardising all tests separately so that there was no main effect of age on cognitive performance nor was there a difference between waves (i.e., the cognitive performance scores at both waves with *M* = 0, *SD* = 1).

**Table 1:** Demographics of participants with IQ data at Wave 1.

	Monozygotic twins (MZ) (*N* = 1,876)	Dizygotic twins (DZ) (*N* = 2,320)	Overall (*N* = 4,196)
Birthweight (g) – mean (SD)	2,360 (530)	2,460 (530)	2,420 (532)
Birthweight (g) difference between twins – median [Min, Max]	200 [0, 1,290]	220 [0, 1,280]	210 [0, 1,290]
Head circumference (cm) – mean (SD)	32.5 (2.12)	32.9 (1.91)	32.7 (2.01)
Head circumference missing data – *N* (%)	1,159 (61.8%)	1,349 (58.1%)	2,508 (59.8%)
Head circumference (cm) – difference between twins – median [Min, Max]	1.00 [0, 10.0]	1.00 [0, 10.0]	1.00 [0, 10.0]
Gestational age (weeks) – median [Min, Max]	36.0 [25.0, 41.0]	37.0 [24.0, 42.0]	37.0 [24.0, 42.0]
Female sex – *N* (%)	1,028 (54.8%)	1,270 (54.7%)	2,298 (54.8%)
Birthweight *Z* scores – mean (SD)	−0.868 (0.871)	−0.762 (0.871)	−0.809 (0.872)
Birthweight *Z* difference between twins –median [Min, Max]	0.487 [0, 3.02]	0.525 [0, 3.36]	0.505 [0, 3.36]
Head circumference *Z* (HCZ) score – mean (SD)	−0.123 (0.983)	−0.0188 (0.980)	−0.0628 (0.982)
HCZ difference between twins – median [Min, Max]	0.619 [0, 3.53]	0.726 [0, 3.38]	0.683 [0, 3.53]
Age Wave 1 (years) – median [Min, Max]	12.1 [4.83, 25.3]	11.7 [4.83, 25.2]	11.8 [4.83, 25.3]
Cognitive performance scores Wave 1 valid twins – *N* (%)	1,876 (100%)	2,320 (100%)	4,196 (100%)
Cognitive performance scores Wave 1 *Z* – mean (SD)	0.050 (0.98)	−0.041 (1.02)	0.00 (1.00)
Age Wave 4 (years) – median [Min, Max]	17.8 [11.0, 30.9]	17.5 [11.0, 30.9]	17.6 [11.0, 30.9]
Cognitive performance scores Wave 4 valid twins – *N* (%)	528 (28.1%)	624 (26.9%)	1,152 (27.5%)
Cognitive performance scores Wave 4 *Z* – mean (SD)	0.068 (0.942)	−0.057 (1.04)	0.00 (1.00)

### Statistical analyses

Each analysis only included twin pairs with complete and plausible data on the birth anthropometrics of interest, gestational age, zygosity, and cognitive performance. Thus, only complete case analyses were performed. To determine how representative these participants were, and to test for selective attrition, we performed two linear mixed models. First, whether twin pairs with valid neonatal data differed on cognitive performance at Wave 1 relative to twin pairs without valid neonatal data. Second, whether twins with valid longitudinal data, differed on cognitive performance at Wave 1 relative to those who could only be included in analyses at Wave 1.

To answer the first research question, each analysis initially examined the linear association of each twin pair's mean birth anthropometric and within‐pair difference (e.g. x¯‐BWZ_Twin_ and Δ‐BWZ_Twin_) with cognitive performance using linear mixed models. Specifically, all linear mixed models account for clustering using a random intercept at the family level while longitudinal models include a further random intercept at the individual level. As these analyses use the co‐twin control design, they decompose the association between BWZ (or HCZ) and cognitive performance into a between and within twin pair component. Of particular interest is the association of the within twin pair differences (Δ‐BWZ_Twin_ or Δ‐HCZ_Twin_) with cognitive performance, as this largely controls for shared environmental and genetic factors. In order to test for nonlinear effects, we replaced the linear terms used in the analyses with a cubic spline function with either two or three knots in the Δ‐BWZ_Twin_/HCZ distributions.

In order to answer the second research question, that the association of the within‐pair difference and cognitive performance diminishes as the average anthropometric value increases, we added an interaction term between each twin pair's mean birth anthropometric and within‐pair difference (i.e. whether Δ‐BWZ_Twin_ is moderated by x¯‐BWZ_Twin_). To answer the third question, the analyses included further tests for moderation of the association of within twin pair differences in anthropometrics and cognitive performance. All models testing for moderation therefore included two‐way interaction terms between Δ‐BWZ_Twin_/HCZ and the potential moderators of gestational age, age at assessment, or zygosity. Whether any effects (i.e. interactions or nonlinear effects) improved model fit relative to the initial linear model was determined via a comparison of the Akaike information criterion (AIC), with the lowest value indicating the most parsimonious model. Furthermore, beta coefficients with *p* values < .05 were considered statistically significant. In order to determine whether the models had sufficient statistical power to determine main effects of x¯‐BWZ_Twin_, Δ‐BWZ_Twin_, or moderation effects, we performed power analyses by simulating data using the SIMR package in R, see Appendix S1 and Table S1 for the results and further details on how these simulations were specified (Green & MacLeod, 2016).

In summary, four initial models were performed using either BWZ or HCZ as the anthropometric of interest and cognitive performance data from only Wave 1 (cross‐sectional) or Waves 1 and 4 combined (longitudinal). These models were then additionally tested for nonlinear and moderation effects, with the optimal model determined by being the most parsimonious, as indicated by the lowest AIC value.

## Results

### Sample characteristics and attrition

In the included sample, there were 4,196 twins with valid cognitive data at Wave 1 as well valid data on birthweight, sex and gestational age, allowing for plausible birthweight *z* scores to be calculated. It was found that twins with valid neonatal data had statistically significantly higher cognitive scores at Wave 1 than those without valid neonatal data, see Table S2. Furthermore, twins with valid longitudinal data (i.e. cognitive data at both waves) had statistically significantly higher cognitive scores at Wave 1 relative to twins who only had cognitive data at Wave 1, see Table S3 and Figure S1 for a cohort flow chart.

The mean within twin difference in birthweight and BWZ scores was 255 g and 0.62z for MZ twins and 282 g and 0.67z for DZ twins. For head circumference, while over 50% of the data was missing, the mean within twin differences in head circumference and HCZ was 0.84 cm and 0.57z for MZ twins and 1.03 cm and 0.73z for DZ twins. Further perinatal and sociodemographic factors by zygosity, specifically for twins with valid cognitive data, are given in Table 1.

### Research questions 1 and 2 – testing for linear and nonlinear associations between birth anthropometrics and cognitive performance scores

An overview of all models is given in Table 2, while the power analyses are shown in Appendix S1 and Table S1. Results indicated that there was sufficient power to determine a main effect of Δ‐BWZ_Twin_ and moderation by age, with power estimates over 98%. In contrast, there was borderline to insufficient power to determine moderation by x¯‐BWZ_Twin_, zygosity or gestational age, with power estimates ranging from 31% to 73%, see Table S1.

**Table 2:** Overview of all co‐twin control models

Model number	Wave 1 or longitudinal	Num observations	Num twin pairs	Anthropometric measure	Δ‐BWZ_Twin_ or Δ‐HCZ_Twin_ Beta	Δ‐BWZ_Twin_ or Δ‐HCZ_Twin_ CI	Nonlinear effects	Any moderation effects	Any moderation smallest *p*	Optimal AIC model
1	Wave 1 Only	4,196	2098	Birthweight Z	.08*	0.04, 0.13	None	Δ‐BWZ_Twin_ X x¯‐BWZ_Twin_	.027*	Model Including Moderation
2	Longitudinal	2,304	576	Birthweight Z	.05	−0.03, 0.14	None	Δ‐BWZ_Twin_ X x¯‐BWZ_Twin_, Δ‐BWZ_Twin_ X Gestational Age	.009*	Model Including Moderation
3	Wave 1 Only	1,368	684	Head Circumference Z	.02	−0.06, 0.10	None	None	.392	Model Including No Moderation
4	Longitudinal	804	201	Head Circumference Z	.01	−0.11, 0.14	None	None	.436	Model Including No Moderation

* *p* < .05.

**Figure 1:**
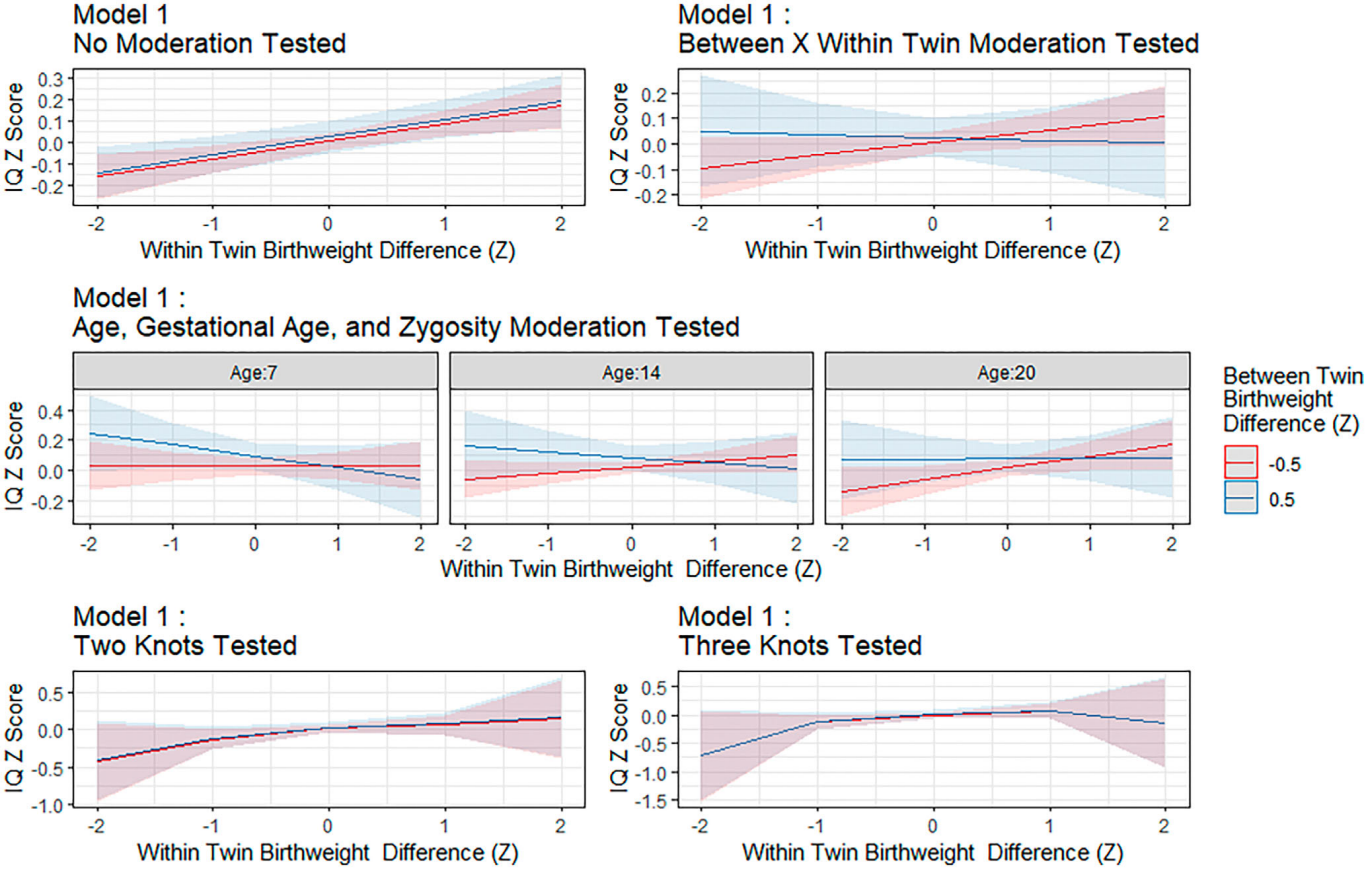
Predicted difference in cognitive performance by within and between twin pair differences in birthweight *z* score – Model 1

In model 1, the association of Δ‐BWZ_Twin_ with cognitive performance scores was 0.08 [95% CI = 0.04–0.13, *p* = .001], see Table S4, Figure 1, and Table 2. In model 2, when reducing the sample to only those with longitudinal data, the association of Δ‐BWZ_Twin_ with cognitive performance was no longer statistically signficant with an association of 0.05 [95% CI = −0.03–0.14, *p* = .187], see Table S4, Figure S2 and Table 2.

In model 3, the association of Δ‐HCZ_Twin_ with cognitive performance scores was 0.02 [95% CI = −0.06–0.10, *p* = .596], see Table S5, Figure S3 and Table 2. Similar, nonsignificant results were found in model 4 when reducing the sample to only those with longitudinal data with an association of 0.01 [95% CI = −0.11–0.14, *p* = .836], see Table S4 and Table 2, see Table S6, Figure S4 and Table 2.

In analyses testing for moderation effects of within and between twin birth anthropometrics, we found a significant interaction of x¯‐BWZ_Twin_ and Δ‐BWZ_Twin_ in both the cross‐sectional and longitudinal analyses, with *β* values equal to −0.08 [95% CI = −0.14 to −0.01, *p* = .027] and −0.14 [95% CI = −0.27 to −0.02, *p* = .024], respectively (Figure 1, Figure S2 and Tables S4, S5). In contrast, when then testing for nonlinear effects of Δ‐BWZ_Twin_ and Δ‐HCZ_Twin_, the addition of either two or three knots never resulted in an improved or more pasimonious model fit as indicated by the AIC value (Table 2).

### Research question 3 – testing for moderation by Zygosity, gestational age, or age at assessment

Including main effects and interaction terms for zygosity, gestational age or age at assessment resulted in improved model fit when BWZ was the anthropometric of interest. This was not the case when HCZ was considered (Table 2). In the longitudinal analysis, the improved model fit was partially attributable to a significant interaction between Δ‐BWZ_Twin_ and gestational age, where the association between Δ‐BWZ_Twin_ and cognitive performance became larger when twins were born at earlier weeks of gestation [*b* = −.05, 95% CI = −0.09 to −0.01, *p* = .009], see Table S5. In contrast, there was no evidence of the association of Δ‐BWZ_Twin_ or Δ‐HCZ_Twin_ with cognitive performance being moderated by zygosity or age at assessment, see Tables S4–S7 and Figures 1 and S2–S4.

## Discussion

Previous research had suggested that difference between twins regarding birthweight (in grams) or relative birthweight (in percentages or *z* scores) were significantly associated with later developmental outcomes, whether that be cognition (Raznahan et al., 2012), attention (Groen‐Blokhuis et al., 2011), or school performance (Torche & Echevarría, 2011). In this study of over 2000 twin pairs, we found evidence that within‐pair differences in relative birthweight was associated with cognitive performance. As there was no evidence for significant moderation by zygosity, this provides further evidence that our findings are not confounded by genetic or other environmental factors that contribute to the similarity of twins. This observational data therefore provides support for theories such as the Developmental Origins of Health and Disease theory, indicating the importance of conditions in the womb for long‐term development (Heindel & Vandenberg, 2015). While improving antenatal conditions is of the utmost importance for reducing the risk of perinatal mortality, the current results suggest there may be further long‐term cognitive benefits of effective interventions in this domain. If one can identify interventions that improve conditions in the womb, which subsequently result in higher birthweights, it may have long‐term benefits by reducing some of the long‐term global burden associated with low birthweight (Liu, Xu, Gong, Zheng, & Chen, 2024).

Measuring head circumference at birth is routine in paediatric care and is thought to act as an indicator of brain growth (Cameron & Schell, 2021). It was therefore investigated whether it would offer similar or superior prediction of later cognitive performance in comparison to birthweight. However, differences between twins regarding head circumference were not significantly associated with later cognitive performance, regardless of whether the analysis was based on longitudinal or cross‐sectional data. As a limitation of our study, approximately 60% of participants had missing data on head circumference at birth, substantially reducing the statistical power. Currently, birthweight is potentially the more straightforward, more often collected indicator of neonatal health which also has more research supporting its association with numerous developmental outcomes (Eves, Nearchou, Wolke, Pluess, & Lemola, 2025). Thus, using low or relatively low birthweight to determine eligibility for later follow‐up or targeted interventions appears to currently have more empirical support in comparison to using head circumference.

While a sample of over 2000 twin pairs is substantial, and approximately double the average sample size in comparison to other twin studies (Polderman et al., 2015), lack of statistical power may have been important for our failure to find nonlinear effects. A recent study, using 30,000 participants from nationally representative samples, found evidence of improved developmental outcomes with increasing relative birthweight until approximately the 69th percentile (*z* = 0.50), followed by a subsequent plateau (Eves et al., 2023). This relatively high percentile may also be rarely observed in twins. It has been reported that twins on average weigh 300 g less than singletons when both are born at term (Hiersch et al., 2020) while the average Fenton birthweight *z* score in the current sample was −0.8 (approximately the 21st percentile). Thus, twins may rarely be born with birthweights large enough to then potentially demonstrate a nonlinear plateauing effect between increasing birthweight and cognitive performance. On the other hand, we did find a consistent interaction of the within and between twin differences in birthweight. This indicated that the within twin difference in birthweight becomes less important when both are born at a higher birthweight, indicative of a plateauing effect. Overall, the reduced sample size and a limited number of infants with relatively high birthweights (i.e. around the 69th percentile or higher), may have hindered replication of the nonlinear associations which had been previously shown in nationally representative samples.

Regarding the investigation into whether relative birthweight is moderated by gestational age, we did find one significant interaction when using longitudinal data. This contradicts a growing body of evidence using general population samples suggesting the association of relative birthweight with cognitive performance is independent of any effect of gestational age. This includes results from longitudinal studies (Eves et al., 2020), nationally representative samples (Eves et al., 2023), and meta‐analyses (Sacchi et al., 2020). Concerning age at assessment, our sample ranged in age from 5 to 31 years old, encompassing a substantial part of the life span. However, it naturally cannot be ruled out that this association may either strengthen or diminish as participants reach middle age and beyond. In addition, the relatively low power to determine interaction effects in this study should be considered. Thus, future studies may look to more robustly investigate whether birthweight is moderated by gestational age or age at assessment via the pooling of either multiple twin studies or using registry data. In addition, studies with fewer missing data on factors such as head circumference or other potentially important antenatal factors, such as whether the twins shared a placenta or not, may provide further evidence for how specific conditions in the womb shape long‐term development.

### Strengths and limitation

Relative to studies using general population sample, the current study has the important advantage of more rigorously controlling for confounders including genetic and shared environmental factors. This provides stronger evidence that conditions in the womb, as indexed by birthweight differences between twins, has a small but significant influence on long‐term cognitive development. After controlling for genetic and shared environmental factors, the standardised effect size of 0.08 is only minimally reduced relative to the effect size of 0.10 reported in nationally representative samples (Eves et al., 2023). Relative to other twin studies, strengths of the current study include studying participants who varied in age, with cognitive performance for many participants assessed in adulthood, and additionally assessing cognitive performance at two timepoints. First, this meant we were able to investigate potential cohort effects, potentially induced by changes in neonatal care (Glass et al., 2015) or rates of twins due to assisted reproductive technologies (Monden, Pison, & Smits, 2021). Second, it allowed for investigation of the longitudinal association between differences in relative birthweight and cognitive performance, allowing for the testing of the theory that this association diminishes when one reaches adulthood (Eryigit Madzwamuse et al., 2015). Limitations include the high rate of missing data and systematic attrition, especially regarding head circumference, and the lack of more detailed pregnancy data, such as whether the twins shared the same placenta. While there was evidence of systematic attrition, especially between the two waves of cognitive assessment, this is unlikely to bias the resulting coefficients from our analyses, as demonstrated in prior work simulating data from longitudinal cohorts (Wolke et al., 2009).

## Conclusion

In conclusion, higher relative birthweight positively predicted higher cognitive performance during the first three decades of life. There was no evidence that this association was nonlinear or that it was moderated by zygosity or age at cognitive assessment. Weight might be a more effective metric, at least for early identification of those at risk of later lower cognitive performance, than head circumference.

## Ethical considerations

Ethical approval was provided by the research ethic committee of the German Psychological Association (DGPS). The dates of approval were the 25th of January 2010 (reference/protocol number: RR 11.2009) and the 25th of September 2013 (reference/protocol number RR 09.2013) (Lang et al., 2020). Regarding informed consent/assent, participants were provided written information regarding the scope and purpose of the study. They were informed of their right to refuse and withdrawal from the study as well as the data protection regulations. Informed consent was obtained from all participants or their legal guardians when participants were under 14 years of age.

**Key points**

**What's known**

- Many past studies using data from the general population report significant associations between neonatal risk factors, such as small for gestational age, and reduced cognitive performance.

**What's new**

- Using data from over 2000 twin pairs, and thus more strictly controlling for genetic and environmental confounding, we found a significant association between differences in relative birthweight and later cognitive performance.

**What's relevant**

- Conditions in the womb, as indexed by twin differences in relative birthweight, is predictive of later differences in cognitive performance.

## Supporting information


**Appendix S1.** Power analysis information.
**Table S1**. Overview of the power analyses for the linear mixed models.
**Table S2**. Differences in IQ at Wave 1 by availability of neonatal data.
**Table S3**. Test for systematic attrition between Wave 1 and Wave 4.
**Table S4**. Model 1.
**Table S5**. Model 2.
**Table S6**. Model 3.
**Table S7**. Model 4.
**Figure S1**. Cohort flow chart.
**Figure S2**. Predicted difference in cognitive performance by within and between twin pair differences in birthweight *z* score – Model 2 (longitudinal).
**Figure S3**. Predicted difference in cognitive performance by within and between twin pair differences in head circumference *z* score – Model 3 (cross‐sectional).
**Figure S4**. Predicted difference in cognitive performance by within and between twin pair differences in head circumference *z* score ‐ Model 4 (longitudinal).

## Data Availability

The data that support the findings of this study are openly available in gesis at: https://www.gesis.org/en/services/crm/data‐application‐form?study_number=ZA6701&study_title=TwinLife&study_doi=10.4232%2F1.14531&study_access_class=C&study_groups=GESIS%20Community%20Data&form_lang=en&no_cache=1. The preregistration and analysis code used in this publication is available here: https://osf.io/j43k8/overview?view_only=b0b873688327434ea8dc7febd321486a.
