# Supplementary material for: Anthropometric differences between twins at birth and their association with later cognitive performance
Source: J Child Psychol Psychiatry. 2026 May 23;67(10):1692–700. doi: 10.1111/jcpp.70170 (PMC13535700; doi:10.1111/jcpp.70170)
Supplement: Supplementary file 1 [file JCPP-67-1692-s001.docx]

**Anthropometric differences between twins at birth and their association with later cognitive performance**

**Supporting Information**

Contents

[Appendix S1. Power Analysis Information	2](#_Toc229410800)

[Table S1- Overview of the power analyses for the linear mixed models	2](#_Toc229410801)

[Table S2 -Differences in IQ at Wave 1 by availability of neonatal data	3](#_Toc229410802)

[Table S3- Test for systematic attrition between wave 1 and wave 4	3](#_Toc229410803)

[Figure S1- Cohort Flow Chart	4](#_Toc229410804)

[Table S4- Model 1	5](#_Toc229410805)

[Table S5 – Model 2	6](#_Toc229410806)

[Table S6- Model 3	7](#_Toc229410807)

[Table S7- Model 4	8](#_Toc229410808)

[Figure S2. Predicted difference in cognitive performance by within and between twin pair differences in birthweight z score - Model 2 (longitudinal).	10](#_Toc229410809)

[Figure S3. Predicted difference in cognitive performance by within and between twin pair differences in head circumference z score - Model 3 (cross sectional).	11](#_Toc229410810)

[Figure S4. Predicted difference in cognitive performance by within and between twin pair differences in head circumference z score - Model 4 (longitudinal).	12](#_Toc229410811)

# Appendix S1. Power Analysis Information

Simulation analyses were conducted where pseudo data was created with similar properties to the TwinLife data. The pseudo data was created with 2098 twin pairs, the same as the largest analysis in the current manuscript. We modelled the pseudo IQ scores so that the within and between twin deviation on birth anthropometrics (x̄-BWZ_Twin_ and Δ-BWZ_Twin_) would be associated with the pseudo IQ score, with beta coefficients equal to 0.1, and an interaction of -0.05. Furthermore, we modelled the data so that the effect of within twin deviation on pseudo IQ differed by zygosity (beta = -0.05), that the effect of within twin deviation on pseudo IQ diminished with each week of gestation (beta = -0.02), and that the effect of within twin deviation on pseudo IQ diminished with age (beta = -0.02). Finally, we modelled the data so that there was an intraclass coefficient of 0.8, meaning pseudo IQ scores would be similar for each twin.

After simulating the data, we ran a linear mixed model in order to determine whether the effects would be deemed statistically significant (p<0.05). We then used the SIMR package, running 6 tests to determine the power of each specific effect of interest. Namely, a main effect of within twin deviation, a main effect of between twin deviation, an interaction of between twin differences and within twin differences, an interaction of within twin differences with age, an interaction of within twin differences with gestational age, and finally an interaction of within twin differences with zygosity. Each power analysis was performed by performing an F test.

# Table S1- Overview of the power analyses for the linear mixed models

| Effect of interest | P value of beta coefficient when using pseudo data | Power for model comparison | Power 95% Confidence Interval |
| --- | --- | --- | --- |
| Mean Twin Birthweight Z (x̄-BWZ_Twin_ ) | 0.108 | 44.00% | (34.08, 54.28) |
| Within Twin Birthweight Z (Δ-BWZ_Twin_) | <0.001 | 98.00% | (92.96, 99.76) |
| x̄-BWZ_Twin X_ Δ-BWZ_Twin_ | 0.166 | 31.00% | (22.13, 41.03) |
| Δ-BWZ_Twin_ × gestational age | 0.047 | 51.00% | (40.80, 61.14) |
| Δ-BWZ_Twin X_ zygosity | 0.042 | 73.00% | (63.20, 81.39) |
| Δ-BWZ_Twin X_ age | <0.001 | 100.0% | (96.38, 100.0) |

# Table S2 -Differences in IQ at Wave 1 by availability of neonatal data

|  | IQ Wave 1 Z | | |
| --- | --- | --- | --- |
| *Predictors* | *Estimates* | *CI* | *p* |
| (Intercept) | -0.06 | -0.11 – -0.02 | 0.004 |
| Plausible Neonatal Data (ref: missing/non plausible neonatal data) | 0.08 | 0.03 – 0.14 | 0.003 |
| Random Effects | | | |
| σ^2^ | 0.44 | | |
| τ_00_ _fid_ | 0.56 | | |
| ICC | 0.56 | | |
| N _fid_ | 2349 | | |
| Observations | 7561 | | |
| Marginal R^2^ / Conditional R^2^ | 0.002 / 0.558 | | |

# Table S3- Test for systematic attrition between wave 1 and wave 4

|  | IQ Wave 1 Z | | |
| --- | --- | --- | --- |
| *Predictors* | *Estimates* | *CI* | *p* |
| (Intercept) | 0.32 | 0.25 – 0.39 | <0.001 |
| Participant only included at Wave 1 (ref: participant assessed at Wave 4) | -0.35 | -0.43 – -0.27 | <0.001 |
| Random Effects | | | |
| σ^2^ | 0.43 | | |
| τ_00_ _fid_ | 0.47 | | |
| ICC | 0.52 | | |
| N _fid_ | 2098 | | |
| Observations | 4196 | | |
| Marginal R^2^ / Conditional R^2^ | 0.026 / 0.533 | | |

# Figure S1- Cohort Flow Chart

# Table S4- Model 1

|  | IQ_Z | | | IQ_Z | | | IQ_Z | | |
| --- | --- | --- | --- | --- | --- | --- | --- | --- | --- |
| *Predictors* | *Estimates* | *CI* | *p* | *Estimates* | *CI* | *p* | *Estimates* | *CI* | *p* |
| (Intercept) | 0.02 | -0.04 – 0.07 | 0.557 | 0.02 | -0.04 – 0.07 | 0.557 | 0.11 | 0.03 – 0.18 | 0.005 |
| Mean Twin Birthweight Z (x̄-BWZTwin ) | 0.02 | -0.03 – 0.07 | 0.420 | 0.02 | -0.03 – 0.07 | 0.420 | 0.06 | 0.01 – 0.12 | 0.025 |
| Within Twin Birthweight Z Difference (Δ-BWZTwin) | 0.08 | 0.04 – 0.13 | 0.001 | 0.02 | -0.06 – 0.10 | 0.623 | -0.01 | -0.11 – 0.10 | 0.915 |
| X̄-BWZTwin :Δ-BWZTwin |  |  |  | -0.06 | -0.12 – -0.00 | 0.046 | -0.08 | -0.14 – -0.01 | 0.027 |
| Zygosity: DZ |  |  |  |  |  |  | -0.11 | -0.18 – -0.03 | 0.007 |
| Gestational Age (weeks) |  |  |  |  |  |  | 0.03 | 0.01 – 0.05 | 0.001 |
| Age (in months) |  |  |  |  |  |  | -0.00 | -0.00 – 0.00 | 0.720 |
| Δ-BWZTwin:ZygosityDZ |  |  |  |  |  |  | 0.03 | -0.07 – 0.13 | 0.560 |
| Δ-BWZTwin:Gestational Age (weeks) |  |  |  |  |  |  | -0.02 | -0.04 – 0.01 | 0.137 |
| Δ-BWZTwin:Age (in months) |  |  |  |  |  |  | 0.00 | -0.00 – 0.00 | 0.107 |
| Random Effects | | | | | | | | | |
| σ^2^ | 0.43 | | | 0.43 | | | 0.43 | | |
| τ_00_ | 0.56 _fid_ | | | 0.56 _fid_ | | | 0.56 _fid_ | | |
| ICC | 0.57 | | | 0.57 | | | 0.56 | | |
| N | 2098 _fid_ | | | 2098 _fid_ | | | 2098 _fid_ | | |
| Observations | 4196 | | | 4196 | | | 4196 | | |
| Marginal R^2^ / Conditional R^2^ | 0.001 / 0.566 | | | 0.002 / 0.566 | | | 0.009 / 0.567 | | |

# Table S5 – Model 2

|  | IQ_Z | | | IQ_Z | | | IQ_Z | | |
| --- | --- | --- | --- | --- | --- | --- | --- | --- | --- |
| *Predictors* | *Estimates* | *CI* | *p* | *Estimates* | *CI* | *p* | *Estimates* | *CI* | *p* |
| (Intercept) | 0.01 | -0.08 – 0.10 | 0.849 | 0.01 | -0.08 – 0.10 | 0.849 | 0.14 | 0.02 – 0.26 | 0.023 |
| Mean Twin Birthweight Z (x̄-BWZTwin ) | 0.01 | -0.07 – 0.10 | 0.799 | 0.01 | -0.07 – 0.10 | 0.799 | 0.06 | -0.03 – 0.15 | 0.222 |
| Within Twin Birthweight Z Difference (Δ-BWZTwin) | 0.05 | -0.03 – 0.14 | 0.187 | -0.01 | -0.14 – 0.12 | 0.894 | -0.09 | -0.26 – 0.08 | 0.318 |
| X̄-BWZTwin:Δ-BWZTwin |  |  |  | -0.07 | -0.18 – 0.04 | 0.212 | -0.14 | -0.27 – -0.02 | 0.024 |
| Zygosity: DZ |  |  |  |  |  |  | -0.17 | -0.29 – -0.05 | 0.006 |
| Gestational Age (weeks) |  |  |  |  |  |  | 0.03 | 0.00 – 0.05 | 0.029 |
| Age (in months) |  |  |  |  |  |  | -0.00 | -0.00 – 0.00 | 0.859 |
| Δ-BWZTwin:ZygosityDZ |  |  |  |  |  |  | 0.04 | -0.12 – 0.21 | 0.622 |
| Δ-BWZTwin:Gestational Age (weeks) |  |  |  |  |  |  | -0.05 | -0.09 – -0.01 | 0.009 |
| Δ-BWZTwin:Age (in months) |  |  |  |  |  |  | 0.00 | -0.00 – 0.00 | 0.205 |
| Random Effects | | | | | | | | | |
| σ^2^ | 0.55 | | | 0.55 | | | 0.55 | | |
| τ_00_ | 0.07 _pid:fid_ | | | 0.07 _pid:fid_ | | | 0.06 _pid:fid_ | | |
|  | 0.38 _fid_ | | | 0.38 _fid_ | | | 0.38 _fid_ | | |
| ICC | 0.45 | | | 0.45 | | | 0.44 | | |
| N | 1152 _pid_ | | | 1152 _pid_ | | | 1152 _pid_ | | |
|  | 576 _fid_ | | | 576 _fid_ | | | 576 _fid_ | | |
| Observations | 2304 | | | 2304 | | | 2304 | | |
| Marginal R^2^ / Conditional R^2^ | 0.001 / 0.451 | | | 0.001 / 0.451 | | | 0.014 / 0.453 | | |

# Table S6- Model 3

|  | IQ_Z | | | IQ_Z | | | IQ_Z | | |
| --- | --- | --- | --- | --- | --- | --- | --- | --- | --- |
| *Predictors* | *Estimates* | *CI* | *p* | *Estimates* | *CI* | *p* | *Estimates* | *CI* | *p* |
| (Intercept) | 0.00 | -0.06 – 0.07 | 0.965 | 0.00 | -0.06 – 0.07 | 0.965 | 0.05 | -0.05 – 0.16 | 0.312 |
| Mean Twin Head Circumference Z  (X̄-HCZTwin) | 0.03 | -0.05 – 0.10 | 0.488 | 0.03 | -0.05 – 0.10 | 0.488 | 0.05 | -0.03 – 0.12 | 0.252 |
| Within Twin Head Circumference  Z Difference (Δ-HCZTwin) | 0.02 | -0.06 – 0.10 | 0.596 | 0.02 | -0.05 – 0.10 | 0.544 | 0.06 | -0.07 – 0.20 | 0.365 |
| X̄-HCZTwin:Δ-HCZTwin |  |  |  | 0.04 | -0.05 – 0.12 | 0.430 | 0.03 | -0.06 – 0.12 | 0.502 |
| Zygosity: DZ |  |  |  |  |  |  | -0.09 | -0.22 – 0.05 | 0.207 |
| Gestational Age (weeks) |  |  |  |  |  |  | 0.03 | -0.00 – 0.06 | 0.072 |
| Age (in months) |  |  |  |  |  |  | -0.00 | -0.00 – 0.00 | 0.739 |
| Δ-HCZTwin:ZygosityDZ |  |  |  |  |  |  | -0.07 | -0.24 – 0.10 | 0.407 |
| Δ-HCZTwin:Gestational Age (weeks) |  |  |  |  |  |  | -0.02 | -0.05 – 0.02 | 0.392 |
| Δ-HCZTwin:Age (in months) |  |  |  |  |  |  | -0.00 | -0.00 – 0.00 | 0.454 |
| Random Effects | | | | | | | | | |
| σ^2^ | 0.46 | | | 0.46 | | | 0.46 | | |
| τ_00_ | 0.54 _fid_ | | | 0.54 _fid_ | | | 0.54 _fid_ | | |
| ICC | 0.54 | | | 0.54 | | | 0.54 | | |
| N | 684 _fid_ | | | 684 _fid_ | | | 684 _fid_ | | |
| Observations | 1368 | | | 1368 | | | 1368 | | |
| Marginal R^2^ / Conditional R^2^ | 0.001 / 0.543 | | | 0.001 / 0.543 | | | 0.006 / 0.545 | | |

# Table S7- Model 4

|  | IQ_Z | | | IQ_Z | | | IQ_Z | | |
| --- | --- | --- | --- | --- | --- | --- | --- | --- | --- |
| *Predictors* | *Estimates* | *CI* | *p* | *Estimates* | *CI* | *p* | *Estimates* | *CI* | *p* |
| (Intercept) | 0.01 | -0.08 – 0.11 | 0.781 | 0.01 | -0.08 – 0.11 | 0.781 | 0.12 | -0.03 – 0.27 | 0.110 |
| Mean Twin Head Circumference Z  (X̄-HCZTwin) | 0.11 | -0.01 – 0.23 | 0.074 | 0.11 | -0.01 – 0.23 | 0.074 | 0.14 | 0.01 – 0.26 | 0.031 |
| Within Twin Head Circumference  Z Difference (Δ-HCZTwin) | 0.01 | -0.11 – 0.14 | 0.836 | 0.01 | -0.12 – 0.14 | 0.863 | 0.04 | -0.17 – 0.26 | 0.680 |
| X̄-HCZTwin:Δ-HCZTwin |  |  |  | -0.07 | -0.23 – 0.10 | 0.429 | -0.07 | -0.23 – 0.10 | 0.436 |
| Zygosity: DZ |  |  |  |  |  |  | -0.18 | -0.38 – 0.01 | 0.068 |
| Gestational Age (weeks) |  |  |  |  |  |  | 0.04 | -0.01 – 0.09 | 0.162 |
| Age (in months) |  |  |  |  |  |  | -0.00 | -0.00 – 0.00 | 0.934 |
| Δ-HCZTwin:ZygosityDZ |  |  |  |  |  |  | -0.06 | -0.33 – 0.21 | 0.674 |
| Δ-HCZTwin:Gestational Age (weeks) |  |  |  |  |  |  | 0.00 | -0.06 – 0.07 | 0.930 |
| Δ-HCZTwin:Age (in months) |  |  |  |  |  |  | 0.00 | -0.00 – 0.00 | 0.967 |
| Random Effects | | | | | | | | | |
| σ^2^ | 0.61 | | | 0.61 | | | 0.61 | | |
| τ_00_ | 0.07 _pid:fid_ | | | 0.07 _pid:fid_ | | | 0.08 _pid:fid_ | | |
|  | 0.31 _fid_ | | | 0.31 _fid_ | | | 0.30 _fid_ | | |
| ICC | 0.39 | | | 0.39 | | | 0.38 | | |
| N | 402 _pid_ | | | 402 _pid_ | | | 402 _pid_ | | |
|  | 201 _fid_ | | | 201 _fid_ | | | 201 _fid_ | | |
| Observations | 804 | | | 804 | | | 804 | | |
| Marginal R^2^ / Conditional R^2^ | 0.008 / 0.391 | | | 0.009 / 0.391 | | | 0.022 / 0.396 | | |

# Figure S2. Predicted difference in cognitive performance by within and between twin pair differences in birthweight z score - Model 2 (longitudinal).

# Figure S3. Predicted difference in cognitive performance by within and between twin pair differences in head circumference z score - Model 3 (cross sectional).

# Figure S4. Predicted difference in cognitive performance by within and between twin pair differences in head circumference z score - Model 4 (longitudinal).
